# Data on the combined effect of atovaquone, mefloquine, and 3-bromopyruvic acid against *Echinococcus multilocularis* protoscoleces

**DOI:** 10.1016/j.dib.2022.108707

**Published:** 2022-10-28

**Authors:** Hirokazu Kouguchi, Shigehiro Enkai, Hiroyuki Matsuyama, Masahito Hidaka, Daniel Ken Inaoka, Kiyoshi Kita, Kinpei Yagi

**Affiliations:** a Department of Infectious Diseases, Hokkaido Institute of Public Health, N19 W12, Kita-Ku, Sapporo, Hokkaido 060-0819, Japan; b Department of Pediatrics, Teikyo University School of Medicine, 2-11-1 Kaga, Itabashi-ku, Tokyo 173-8605, Japan; c Department of Molecular Infection Dynamics, Shionogi Global Infectious Diseases Division, Institute of Tropical Medicine (NEKKEN), Nagasaki University, Nagasaki 852-8523, Japan; d School of Tropical Medicine and Global Health, Nagasaki University, 1-12-4 Sakamoto, Nagasaki 852-8523, Japan; e Department of Host-Defense Biochemistry, Institute of Tropical Medicine (NEKKEN), Nagasaki University, 1-12-4 Sakamoto, Nagasaki 852-8523, Japan; f Laboratory of Parasitology, Department of Disease Control, Graduate School of Infectious Diseases, Faculty of Veterinary Medicine, Hokkaido University, N18 W9, Kita-ku, Sapporo, Hokkaido 060-0818, Japan

**Keywords:** *Echinococcus multilocularis*, Echinococcosis, Atovaquone, Mefloquine, 3-Bromopyruvic acid, Drug target

## Abstract

The dataset presented here is related to a previous research article titled “Mitochondrial Complex III in Larval Stage of *Echinococcus multilocularis* as a Potential Chemotherapeutic Target and in vivo Efficacy of Atovaquone Against Primary Hydatid Cysts”[1]. In this report, data were collected from aerobic and anaerobic culture assays of *E. multilocularis* protoscoleces in the presence of three anti-echinococcal drug candidates (atovaquone, mefloquine, and 3-bromopyruvic acid). The data were analyzed for viability of the protoscoleces between day 0 and day 7 upon adding drug candidates. In aerobic condition, all drug candidates caused damage to the protoscoleces, as described previously [1], [2], [3], [4], [5], [6]. Mefloquine, alone as well as in combination with atovaquone, immediately eliminated the protoscoleces, whereas combination of atovaquone with 3-bromopyruvic acid did not show clear synergy. In anaerobic condition, mefloquine, alone as well as in combination with atovaquone, eliminated protoscoleces immediately. 3-Bromopyruvic acid showed stronger efficacy in anaerobic condition than in aerobic condition. Combination of atovaquone with 3-bromopyruvic acid eliminated the protoscoleces, indicating that synergy occurred only under anaerobic condition. The data clarified that combined use of the three drugs eliminated protoscoleces in both aerobic and anaerobic conditions, hence suggesting that these could inhibit aerobic and anaerobic respiration pathways of *Echinococcus multilocularis* in vivo. The obtained data would be useful for the development of new drug dosing method for alveolar echinococcosis.

**Specifications Table**

**Table:** 

Subject	Drug Discovery
Specific subject area	Mitochondrial respiratory system
Type of data	Figure
How the data were acquired	Culture treatment assays using *E. multilocularis* protoscoleces were performed under aerobic and anaerobic conditions. The protoscoleces were treated with drug candidates in culture medium, then stained with trypan blue between day 0 and day 7, and subsequently counted by microscopy.
Data format	Raw Analyzed
Description of data collection	*E. multilocularis* protoscoleces were treated with three anti-echinococcal drug candidates (atovaquone, mefloquine, and 3-bromopyruvic acid) at a final concentration of 50 μM in each culture medium. Viability of protoscoleces was determined by microscopic analysis of more than 120 protoscoleces per well to exclude the contribution of trypan blue.
Data source location	Institution: Hokkaido Institute of Public Health City: Sapporo Country: Japan Latitude and longitude (and GPS coordinates) for collected samples/data: 43°04′58.804′'N; 141°19′59.769′'E
Data accessibility	https://data.mendeley.com/datasets/8gx4p2x5vw/1
Related research article	

## Value of the Data

• Culture treatment assays clarified that the combined use of atovaquone with mefloquine or 3-bromopyruvic acid can eliminate *E. multilocularis* protoscoleces under both aerobic and anaerobic conditions.
• The data suggested that combination of the drugs can exhibit therapeutic efficacy against hydatid cyst in vivo.
• Currently, albendazole is the only therapeutic agent for patients with alveolar echinococcosis; therefore, new anti-echinococcus drugs would need to be developed. The current data provided new insights for the development of anti-echinococcal combination drugs.

## 1 Objective

*E. multilocularis* uses both aerobic and anaerobic respiratory chains for survival in the host animal. We had previously reported that atovaquone alone can inhibit lesion development in mice experimentally infected with *E. multilocularis* [1]. Further, using culture assay, we had shown that atovaquone inhibits the aerobic respiratory chain, but not the anaerobic respiratory chain, of *E. multilocularis*. To augment the therapeutic effect of atovaquone, drug combinations that can eliminate *E. multilocularis* under anaerobic conditions were tested by culture treatment assays. Data provided here showed that the effects of mefloquine and 3-bromopyruvic acid are potentiated when combined with atovaquone.

## 2 Data Description

**Fig. 1:**
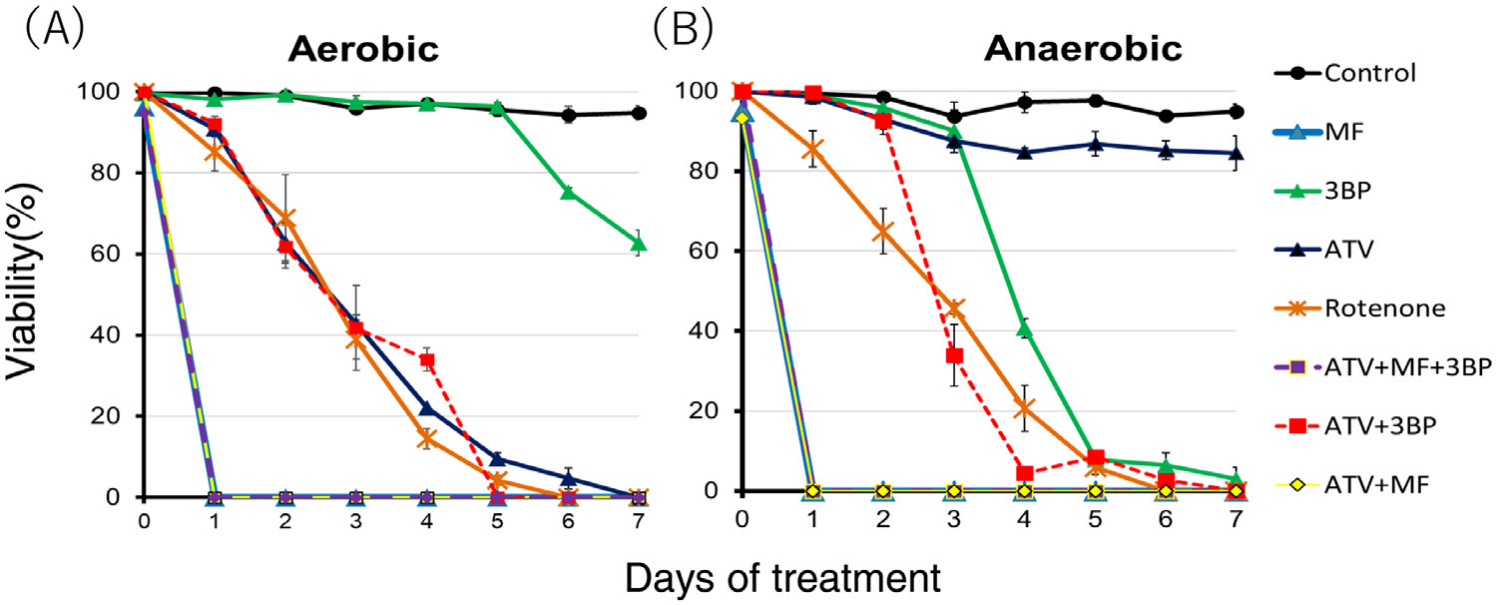
Culture treatment assays under aerobic (A) and anaerobic (B) conditions (oxygen concentration < 0.3%); *E. multilocularis* protoscoleces were treated with atovaquone, mefloquine, 3-bromopyruvic acid, and their combination at a final concentration of 50 μM in each culture medium. Viability of protoscoleces was evaluated by their ability to exclude trypan blue. Protoscoleces were counted in triplicate from day 0 to day 7. Statistical analysis was performed by mean of viability (%), and the standard deviation of triplicate samples was indicated by error bars. Rotenone was added as a positive control. Raw data tables are available as supplementary material.

Fig. 1A shows the anti-parasitic effect of 50 μM atovaquone (ATV), rotenone, mefloquine (MF), 3-bromopyruvic acid (3BP), and combinations of ATV with other two drugs in an aerobic culture experiment using *E. multilocularis* protoscoleces. Rotenone was added as a positive control for drug treatment, as reported previously [1,2]. ATV killed all protoscoleces on day 7, as reported earlier, whereas the addition of MF, alone or in combination (such as ATV + MF or ATV + MF + 3BP), killed all protoscoleces on day 1 itself. Addition of 3BP alone killed 37% of the protoscoleces on day 7, whereas addition of ATV with 3BP resulted in a similar percentage reduction in viability as with ATV alone. Combination of ATV and 3BP killed all protoscoleces on day 5, 2 days earlier than that by ATV alone.

Fig. 1B shows the results of culture assay under anaerobic conditions. ATV showed little anti-parasitic effect against protoscoleces, as reported previously [1]. When MF, alone and in combination (ATV + MF or ATV + MF + 3BP), was added, all protoscoleces were killed on day 1 as in aerobic condition. 3BP killed more than 90% of protoscoleces on day 5, and its effect was more pronounced than that under aerobic conditions. The combination of ATV and 3BP killed all protoscoleces on day 7 under anaerobic conditions, the decrease in viability being faster than that with 3BP alone.

All raw data tables described here are available as supplementary material.

## 3 Experimental Design, Materials and Methods

### 3.1 Preparation of the *E. multilocularis* protoscoleces

*E. multilocularis* (Nemuro strain), maintained in the Hokkaido Institute of Public Health, was used in this study. Cotton rats (*Sigmodon hispidus)* were experimentally infected by oral administration of 200 eggs of *E. multilocularis* and maintained for more than 4 months to obtain mature protoscoleces. The infected cotton rats were sacrificed, following isoflurane overdose, to obtain the cyst tissues containing *E. multilocularis* protoscoleces. The cyst tissues were minced with scissors, passed through a metal mesh, shredded completely, and then repeatedly suspended and washed with PBS in a tall beaker to isolate protoscoleces based on the difference in buoyancy between the protoscoleces and other tissues. The isolated protoscoleces were transferred to petri dishes and gently turned in PBS to remove calcareous corpuscles [1,2].

### 3.2 Culture treatment assay using *E. multilocularis* protoscoleces

The obtained protoscoleces were cultured in Connaught Medical Research Laboratories 1066 medium (Gibco, Grand Island, NY, USA) containing 23 mM 4-(2-hydroxyethyl)-1-piperazine ethanesulfonic acid, 0.5% (w/v) D (+)-glucose, 0.4 mM sodium taurocholate (Wako Pure Chemical Industries), 0.5% (w/v) yeast extract (Difco Laboratories, Detroit, MI, USA), 57 mM sodium hydrogen carbonate, 2 mM L-glutamine (Gibco), 100 U/mL penicillin, and 100 μg/mL streptomycin (Gibco) in a six-well plate. Half of the medium was replaced on day 3. For aerobic cultures, the six-well plate was incubated at 37°C in a CO_2_ incubator with 5% CO_2_. For anaerobic cultures, six-well plates were sealed in plastic containers with oxygen-detecting agents and oxygen scavengers (Aneromeito®, Nissui Pharmaceutical, Tokyo, Japan) to maintain the oxygen concentration under 0.3% at 37°C. To assess the effect of drug candidates against *E. multilocularis* protoscoleces, the parasites were treated with ATV, MF, 3BP, and their combination at a final concentration of 50 μM each in the culture medium. Rotenone was used as the control for comparison with previous results [1,2]. The control group was supplemented with 0.5% (v/v) dimethyl sulfoxide (DMSO), and all conditions were assayed in triplicate. Viability of protoscoleces was determined by microscopic observation of more than 120 protoscoleces per well using the trypan blue exclusion test. The protoscoleces were observed daily for 7 consequent days.

## Ethics Statements

This study was performed in strict accordance with the National Institutes of Health guide for the care and use of Laboratory animals, and the ethics committee of the Hokkaido Institute of Public Health approved the protocol for the animal experiments (permit number: K25-2 and K22-1). All surgeries were performed under sodium pentobarbital anesthesia, and every effort was made to minimize suffering of the animals.

## Funding

This work was supported by 10.13039/501100001691JSPS KAKENHI [grant numbers JP20K06402 and JP22K07048].

## CRediT authorship contribution statement

**Hirokazu Kouguchi:** Conceptualization, Data curation, Writing – original draft. **Shigehiro Enkai:** Methodology, Writing – review & editing. **Hiroyuki Matsuyama:** Formal analysis. **Masahito Hidaka:** Investigation. **Daniel Ken Inaoka:** Writing – review & editing. **Kiyoshi Kita:** Resources, Supervision. **Kinpei Yagi:** Supervision.

## Declaration of Competing Interest

The authors declare that they have no known competing financial interests or personal relationships that could have appeared to influence the work reported in this paper.

## Data Availability

The row data presented in Data of combination effect of atovaquone, mefloquine and 3-bromopyruvic acid against Echinococcus multilocularis protoscoleces. (Original data) (Mendeley Data).
